# Supplementary material for: Comprehensive Analysis for Anti-Cancer Target-Indication Prioritization of Placental Growth Factor Inhibitor (PGF) by Use of Omics and Patient Survival Data
Source: Biology (Basel). 2023 Jul 7;12(7):970. doi: 10.3390/biology12070970 (PMC10376188; doi:10.3390/biology12070970)
Supplement: Supplementary file 1 [file biology-12-00970-s001.zip › biology-2441075-supplementary.pdf]

Supplementary Materials

Table S1. Gene Ontology summary of PGF

Category domain	GO Term	Evidence Code	Source
Biological Process	positive regulation of endothelial cell proliferation	IBA	PMID:21873635
	signal transduction	TAS	PMID:9467961
	cell-cell signaling	TAS	PMID:9467961
	positive regulation of mast cell chemotaxis	IBA	PMID:21873635
	positive chemotaxis	IEA	GO_REF:0000108
	positive regulation of protein phosphorylation	IBA	PMID:21873635
	sprouting angiogenesis	IBA	PMID:21873635
	vascular endothelial growth factor signaling pathway	IBA	PMID:21873635
	vascular endothelial growth factor receptor signaling pathway	IBA	PMID:21873635
	female pregnancy	IEA	GO_REF:0000107
	positive regulation of angiogenesis	IBA	PMID:21873635
	response to hypoxia	IBA	PMID:21873635
	response to xenobiotic stimulus	IEA	GO_REF:0000107
	animal organ regeneration	IEA	GO_REF:0000107
	cellular response to hormone stimulus	IEA	GO_REF:0000107
	cell differentiation	IEA	GO_REF:0000043
	positive regulation of cell division	IEA	GO_REF:0000043
	induction of positive chemotaxis	IBA	PMID:21873635
	positive regulation of cell population proliferation	IMP	PMID:21215706
Cellular Component	extracellular space	IBA	PMID:21873635
	extracellular region	TAS	Reactome:R-HSA-194311
	extracellular region	TAS	Reactome:R-HSA-195378
	membrane	IEA	GO_REF:0000002
Molecular Function	growth factor activity	IBA	PMID:21873635
	protein binding	IPI	PMID:20660291
	growth factor activity	IMP	PMID:21215706

	vascular endothelial growth factor receptor binding	IBA	PMID:21873635
	heparin binding	IEA	GO_REF:0000043
	identical protein binding	IEA	GO_REF:0000107
	chemoattractant activity	IBA	PMID:21873635
	protein-containing complex binding	IEA	GO_REF:0000107
	protein binding	IPI	PMID:14684734

Figure S1. VEGF/PGF signaling interactions

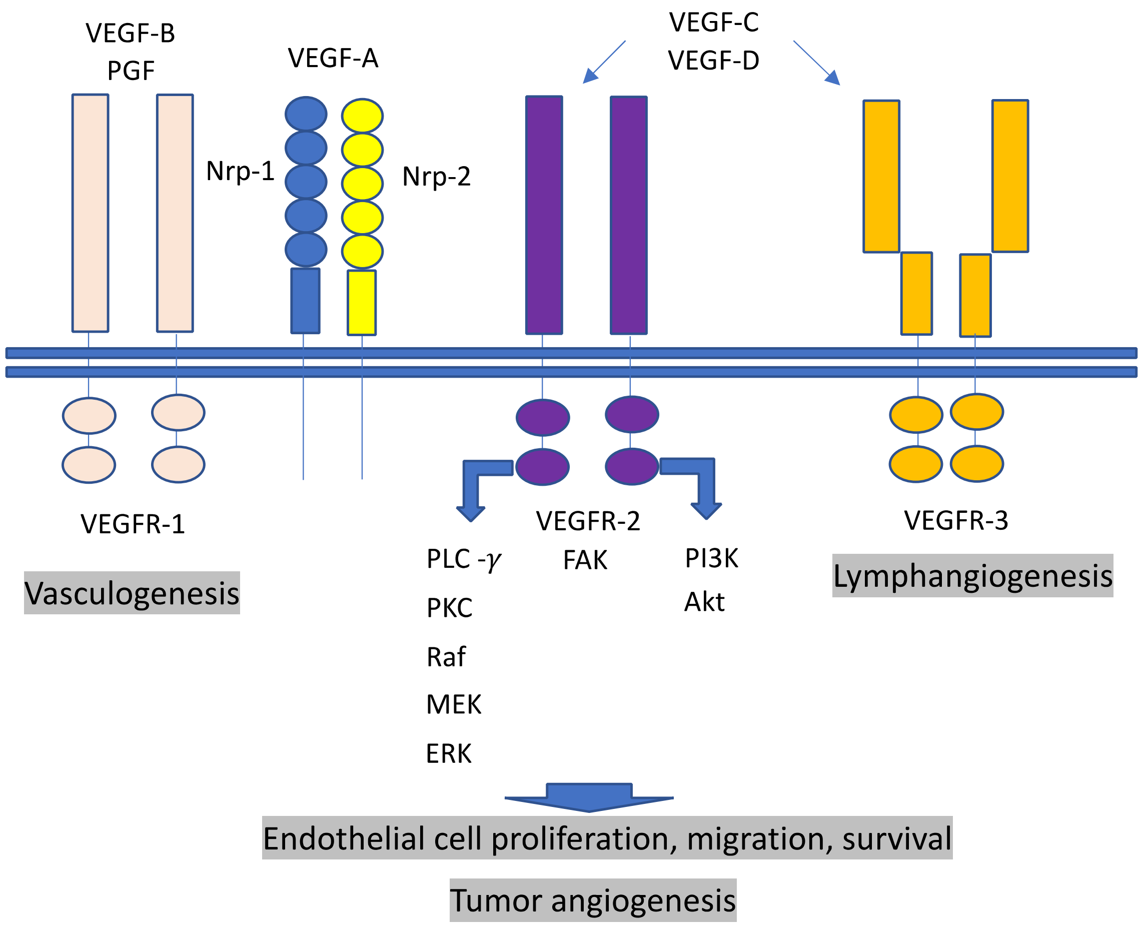

Figure S2. Protein-Protein Interaction to PGF.
At protein level, PGF is functionally interacting with VEGFA, PIK3R1, PLCG1, NRP1, FLT1(VEGFR1), FLT4(VEGFR2), NTRK1, KDR, PIK3CA and CSF1R.

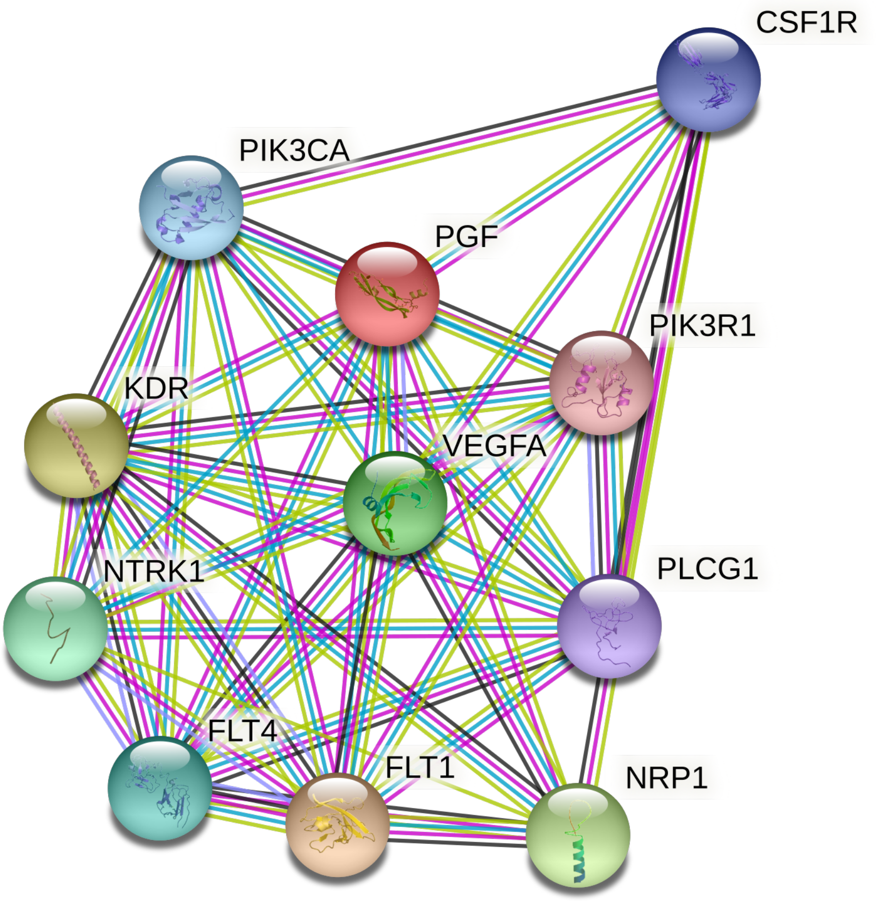

Node	Domain summary
CSF1R	Macrophage colony-stimulating factor 1 receptor; Tyrosine-protein kinase that acts as cell-surface receptor for CSF1 and IL34 and plays an essential role in the regulation of survival, proliferation and differentiation of hematopoietic precursor cells, especially mononuclear phagocytes, such as macrophages and monocytes. Promotes the release of proinflammatory chemokines in response to IL34 and CSF1, and thereby plays an important role in innate immunity and in inflammatory processes.

FLT1	Vascular endothelial growth factor receptor 1; Tyrosine-protein kinase that acts as a cell-surface receptor for VEGFA, VEGFB and PGF, and plays an essential role in the development of embryonic vasculature, the regulation of angiogenesis, cell survival, cell migration, macrophage function, chemotaxis, and cancer cell invasion. May play an essential role as a negative regulator of embryonic angiogenesis by inhibiting excessive proliferation of endothelial cells.
FLT4	Vascular endothelial growth factor receptor 3; Tyrosine-protein kinase that acts as a cell-surface receptor for VEGFC and VEGFD, and plays an essential role in adult lymphangiogenesis and in the development of the vascular network and the cardiovascular system during embryonic development. Promotes proliferation, survival and migration of endothelial cells, and regulates angiogenic sprouting. Signaling by activated FLT4 leads to enhanced production of VEGFC, and to a lesser degree VEGFA, thereby creating a positive feedback loop that enhances FLT4 signaling.
KDR	Vascular endothelial growth factor receptor 2; Tyrosine-protein kinase that acts as a cell-surface receptor for VEGFA, VEGFC and VEGFD. Plays an essential role in the regulation of angiogenesis, vascular development, vascular permeability, and embryonic hematopoiesis. Promotes proliferation, survival, migration and differentiation of endothelial cells. Promotes reorganization of the actin cytoskeleton. Isoforms lacking a transmembrane domain, such as isoform 2 and isoform 3, may function as decoy receptors for VEGFA, VEGFC and/or VEGFD.
NRP1	Neuropilin-1; The membrane-bound isoform 1 is a receptor involved in the development of the cardiovascular system, in angiogenesis, in the formation of certain neuronal circuits and in organogenesis outside the nervous system. It mediates the chemorepulsant activity of semaphorins. It binds to semaphorin 3A, The PLGF-2 isoform of PGF, The VEGF165 isoform of VEGFA and VEGFB. Coexpression with KDR results in increased VEGF165 binding to KDR as well as increased chemotaxis. Regulate VEGF-induced angiogenesis.
NTRK1	High affinity nerve growth factor receptor; Receptor tyrosine kinase involved in the development and the maturation of the central and peripheral nervous systems through regulation of

	proliferation, differentiation and survival of sympathetic and nervous neurons. High affinity receptor for NGF which is its primary ligand. Can also bind and be activated by NTF3/neurotrophin-3. However, NTF3 only supports axonal extension through NTRK1 but has no effect on neuron survival (By similarity).
PGF	Placental growth factor; Placenta growth factor; Growth factor active in angiogenesis and endothelial cell growth, stimulating their proliferation and migration. It binds to the receptor FLT1/VEGFR-1. Isoform PlGF-2 binds NRP1/neuropilin-1 and NRP2/neuropilin-2 in a heparin-dependent manner.
PIK3CA	Phosphatidylinositol 4,5-bisphosphate 3-kinase catalytic subunit alpha isoform; Phosphoinositide-3-kinase (PI3K) that phosphorylates PtdIns (Phosphatidylinositol), PtdIns4P (Phosphatidylinositol 4- phosphate) and PtdIns(4,5)P2 (Phosphatidylinositol 4,5- bisphosphate) to generate phosphatidylinositol 3,4,5-trisphosphate (PIP3). PIP3 plays a key role by recruiting PH domain-containing proteins to the membrane, including AKT1 and PDPK1, activating signaling cascades involved in cell growth, survival, proliferation, motility and morphology.
PIK3R1	Phosphoinositide-3-kinase regulatory subunit alpha/beta/delta; Phosphatidylinositol 3-kinase regulatory subunit alpha; Binds to activated (phosphorylated) protein-Tyr kinases, through its SH2 domain, and acts as an adapter, mediating the association of the p110 catalytic unit to the plasma membrane. Necessary for the insulin-stimulated increase in glucose uptake and glycogen synthesis in insulin-sensitive tissues. Plays an important role in signaling in response to FGFR1, FGFR2, FGFR3, FGFR4, KITLG/SCF, KIT, PDGFRA and PDGFRB.
PLCG1	1-phosphatidylinositol 4,5-bisphosphate phosphodiesterase gamma-1; Mediates the production of the second messenger molecules diacylglycerol (DAG) and inositol 1,4,5-trisphosphate (IP3). Plays an important role in the regulation of intracellular signaling cascades. Becomes activated in response to ligand- mediated activation of receptor-type tyrosine kinases, such as PDGFRA, PDGFRB, FGFR1, FGFR2, FGFR3 and FGFR4.

VEGFA	Vascular endothelial growth factor A; Growth factor active in angiogenesis, vasculogenesis and endothelial cell growth. Induces endothelial cell proliferation, promotes cell migration, inhibits apoptosis and induces permeabilization of blood vessels. Binds to the FLT1/VEGFR1 and KDR/VEGFR2 receptors, heparan sulfate and heparin. NRP1/Neuropilin-1 binds isoforms VEGF-165 and VEGF-145. Isoform VEGF165B binds to KDR but does not activate downstream signaling pathways, does not activate angiogenesis and inhibits tumor growth.
